# Uncovering the Effect of SARS-CoV-2 on Liver Metabolism via Genome-Scale Metabolic Modeling for Reprogramming and Therapeutic Strategies

**DOI:** 10.1021/acsomega.4c00392

**Published:** 2024-03-22

**Authors:** Mustafa Sertbas, Kutlu O. Ulgen

**Affiliations:** Department of Chemical Engineering, Bogazici University, 34342 Istanbul, Turkey

## Abstract

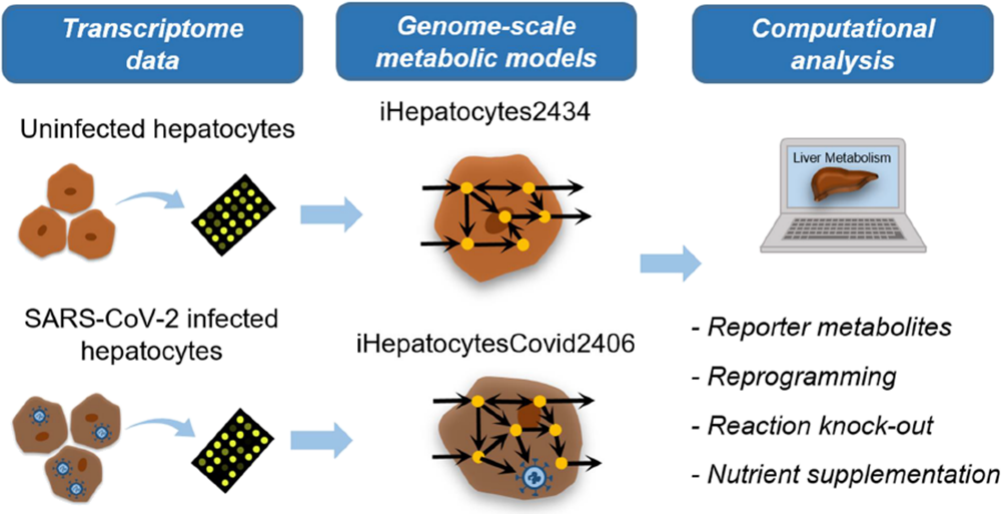

Genome-scale metabolic models (GEMs) are promising computational tools that contribute to elucidating host–virus interactions at the system level and developing therapeutic strategies against viral infection. In this study, the effect of severe acute respiratory syndrome coronavirus 2 (SARS-CoV-2) on liver metabolism was investigated using integrated GEMs of human hepatocytes and SARS-CoV-2. They were generated for uninfected and infected hepatocytes using transcriptome data. Reporter metabolite analysis resulted in significant transcriptional changes around several metabolites involved in xenobiotics, drugs, arachidonic acid, and leukotriene metabolisms due to SARS-CoV-2 infection. Flux balance analysis and minimization of metabolic adjustment approaches unraveled possible virus-induced hepatocellular reprogramming in fatty acid, glycerophospholipid, sphingolipid cholesterol, and folate metabolisms, bile acid biosynthesis, and carnitine shuttle among others. Reaction knockout analysis provided critical reactions in glycolysis, oxidative phosphorylation, purine metabolism, and reactive oxygen species detoxification subsystems. Computational analysis also showed that administration of dopamine, glucosamine, D-xylose, cysteine, and (*R*)-3-hydroxybutanoate contributes to alleviating viral infection. In essence, the reconstructed host–virus GEM helps us understand metabolic programming and develop therapeutic strategies to battle SARS-CoV-2.

## Introduction

The infectious coronavirus disease 2019 (COVID-19) is caused by the severe acute respiratory syndrome coronavirus 2 (SARS-CoV-2) virus and has affected millions of people around the world since its outbreak in December 2019. World Health Organization declared a global emergency on January 30, 2020 and a global pandemic on March 11, 2020.^1^ COVID-19 patients showed viral pneumonia symptoms including fever, fatigue, dry cough, and lymphopenia. In addition to lungs, it may also affect various body parts such as liver, heart, kidneys, blood, and immune system. Comorbid diseases in these organs and malignant tumors increase the severity of illness, especially in older patients. Along with treating COVID-19, extra attention should be paid to the evaluation and treatment of comorbidities.

Computational approaches contribute to understanding the effect of the SARS-CoV-2 virus on host cell metabolism and the development of therapeutic strategies against COVID-19.^2,3^ An important approach is computational drug repurposing, which aims to identify new uses of existing drugs for the SARS-CoV-2 virus. It takes full advantage of drug databases, crystal structures of viral proteins, dynamic molecular simulations, and docking tools; therefore, it considerably reduces the time and expenditure compared to the *de novo* drug design process. Another key approach is computational systems biology, where complex biological systems including host–virus metabolism and their interactions are investigated comprehensively. Multiomics tools such as transcriptomics, proteomics, and metabolomics provide invaluable information about the host–virus metabolic network in healthy and infected cells since they quantify thousands of gene, protein, and metabolite levels, respectively. The analysis of lung transcriptome data revealed significantly altered biological processes including hypoxia response, lung development, respiratory processes, and surfactant metabolism in COVID-19 patients.^4^ Drug enrichment analysis estimated lung surfactants, respiratory stimulants, immunostimulators like sargramostim, and inhibitor drug oseltamivir as potential treatments. Differential transcriptome analysis of liver tissue samples from severe COVID-19 patients resulted in significant upregulation of transcripts in tissue remodeling, G-coupled protein receptor signaling, and transmembrane transport and downregulation in metabolic pathways and mitochondrial functions.^5^

Genome-scale metabolic models (GEMs) have become an important tool in investigating prokaryotic and eukaryotic cells over the past two decades.^6^ GEMs convert the metabolic reaction network inside the cell of interest in conjunction with their related genes and metabolites into a mathematical matrix format. The effect of a certain perturbation on a specific tissue due to a metabolic disease can be computationally estimated. Flux balance analysis (FBA) is the most commonly used constraint-based modeling approach to predict the probable response of cell metabolism via GEMs.^7^ Several GEMs were reconstructed and updated for humans, pathogens, and specific tissues such as liver and brain.^8−12^ Generic human GEMs consist of all metabolic reactions occurring in different human tissues. Along with the multiomics data, generic human GEMs are the key components of automatic reconstruction of tissue-specific GEMs.^13^ Manual reconstruction of tissue-specific GEMs requires obtaining the reactions available in the tissue of interest from the literature. Hepatonet1, one of the first GEMs for human hepatocytes with 2539 reactions and 777 metabolites, was manually reconstructed with experimentally supported reactions occurring in human hepatocytes or liver tissue.^14^ Context-specific GEM extraction algorithms considerably reduce the model reconstruction time from several months to a few minutes. Agren et al. (2014) developed task-driven Integrative Network Inference for Tissues (tINIT) algorithm based on predefined metabolic tasks, multiomics evidence, and Human Metabolic Reaction database 2.0 for automated generation of functional GEMs.^8,15^ Functional personalized GEMs were generated for hepatocellular carcinoma patients and healthy cell types, and potential antimetabolites such as the analogues of l-carnitine, lysine, and methionine were estimated to inhibit hepatocellular carcinoma tumors.

Genome-scale metabolic networks have great potential to investigate host–pathogen interaction by integrated modeling of a specific human tissue and the pathogen of interest.^9,16^ Human alveolar macrophage model (iAB-AMØ-1410) was combined with *Mycobacterium tuberculosis* model (iNJ661) to study the integrated analysis of metabolic changes in the host–pathogen network during infection.^17,18^ In the pathogen part, shifts in carbon uptake and central metabolism were observed with the production of acetyl-CoA from fatty acids through a glyoxylate shunt. In the alveolar macrophage part, nitric oxide production was increased, but ATP production and nucleotide synthesis were decreased. In another study, *Salmonella*-infected HeLa cells were investigated using integrated pathogen-host genome-scale metabolic network and dual transcriptome data.^19^ Subsequent to drug target prioritization procedure, pabB was selected as the putative drug target, and docking simulations were performed to predict candidate compounds inhibiting pabB. Similar to bacterial pathogens, GEMs are also used in viral infection modeling in which the virus is represented by a pseudoreaction called virus biomass objective function (VBOF) based on its constituents.^20,21^ Specific alterations were predicted in central carbon metabolism and lipid biosynthesis pathways and also a set of host reactions inhibiting virus production in the integrated host–virus metabolic network of human macrophage and chikungunya, dengue, and zika viruses.^20^ iAB-AMØ-1410 was used as the human macrophage metabolic network,^17^ and VBOFs were generated for three viruses.

With the emergence of COVID-19, several human-virus GEM studies were performed for the analysis of SARS-CoV-2 virus effect on host cell metabolism and potential drug targets.^21^ Integrated human alveolar macrophage and SARS-CoV-2 GEM were manually reconstructed by incorporating VBOF based on amino acids, nucleotides, and energy requirements of SARS-CoV-2 into iAB-AMØ-1410.^22^ Host and virus optimization analyses were performed by using FBA and each biomass reaction as an objective function. Reaction knockout approach resulted in guanylate kinase (GK1) reaction as a promising metabolic target for antiviral strategies since inhibition of GK1 decreases the virus biomass flux to zero. This study was further extended to different SARS-CoV-2 variants by calculating the stoichiometric coefficients of VBOF for each mutation.^23^ GK1 was confirmed as a potential antiviral target with the new targets as purine, pyrimidine, and lipid metabolisms. In addition to the severe effect of COVID-19 on lungs, its infection also damages other organs, including liver.

In this study, the effect of COVID-19 on liver metabolism is investigated by integrated genome-scale metabolic modeling of human hepatocytes and the SARS-CoV-2 virus interaction. GEMs were generated for uninfected and infected hepatocytes by using transcriptome data and the tINIT algorithm. Reporter metabolite analysis resulted in significant transcriptional changes around several metabolites involved in different subsystems such as xenobiotics, drug, arachidonic acid, leukotriene, and linoleate metabolisms. FBA and the minimization of metabolic adjustment (MOMA) approaches were employed to simulate metabolic fluxes and reveal possible hepatocellular rewiring metabolism due to COVID-19 infection. Reaction knockout analysis identified critical reactions to inhibit virus growth and suggest treatment strategies against SARS-CoV-2.

## Results and Discussion

### Reconstructed Uninfected and SARS-CoV-2-Infected GEMs

**Figure 1:**
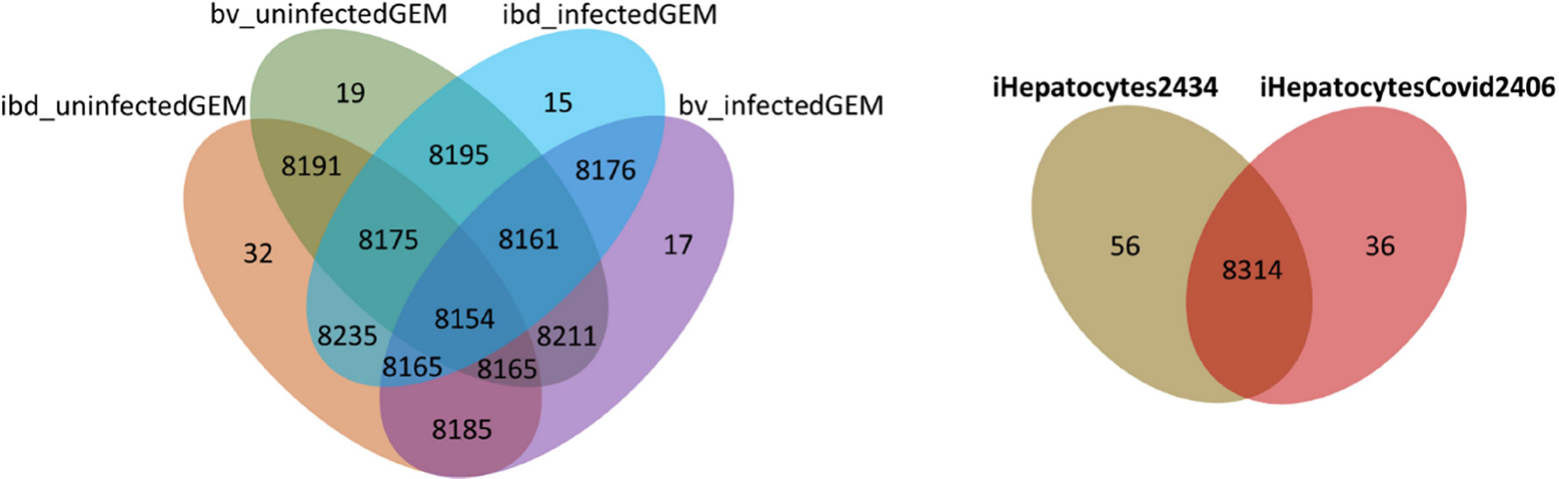
Number of shared reactions in uninfected and SARS-CoV-2-infected GEMs.

For the investigation of uninfected (normal physiological state) liver metabolism, two GEMs, named, ibd_uninfectedGEM and bv_uninfectedGEM (Table 1), were generated based on transcriptome data for the hepatic region around the bile duct and blood vessel by using the tINIT algorithm. These two metabolic networks were merged to come up with a new liver-specific GEM (iHepatocytes2434) with 8370 metabolic reactions, 2434 genes, and 5851 metabolites for the investigation of liver metabolism at the resting condition (Supplementary data 1). Similarly, ibd_infectedGEM and bv_infectedGEM were extracted from Human1 based on SARS-CoV-2-infected transcriptome data. In addition to 57 essential metabolic tasks, SARS-CoV-2 biomass reaction, representing the component of the virus (obtained from Leonidou et al., 2023), was used as an essential task in the tINIT algorithm. The combined SARS-CoV-2- infected liver-specific GEM (iHepatocytesCovid2406) resulted in 8350 metabolic reactions, 2406 genes, and 5843 metabolites to elucidate liver-virus interactions (Supplementary data 1). The initial uninfected and infected genome-scale networks have 8154 mutual reactions, and the final iHepatocytes2434 and iHepatocytesCovid2406 metabolic networks include 8314 shared reactions (Figure 1). Metabolic task performance of both genome-scale models resulted in 240 successfully performed metabolic tasks among 257 tasks listed by Robinson et al. (2020).^11^

**Table 1:** Comparison of the Number of Reactions, Metabolites, and Genes in Uninfected and SARS-CoV-2-Infected Liver-Specific GEMs

genome-scale metabolic models	reactions	metabolites	genes
ibd_uninfectedGEM	8292	5827	2376
bv_uninfectedGEM	8269	5811	2334
***iHepatocytes2434***	**8370**	**5851**	**2434**
ibd_infectedGEM	8274	5822	2366
bv_infectedGEM	8252	5807	2316
***iHepatocytesCovid2406***	**8350**	**5843**	**2406**

### Reporter Metabolites

**Figure 2:**
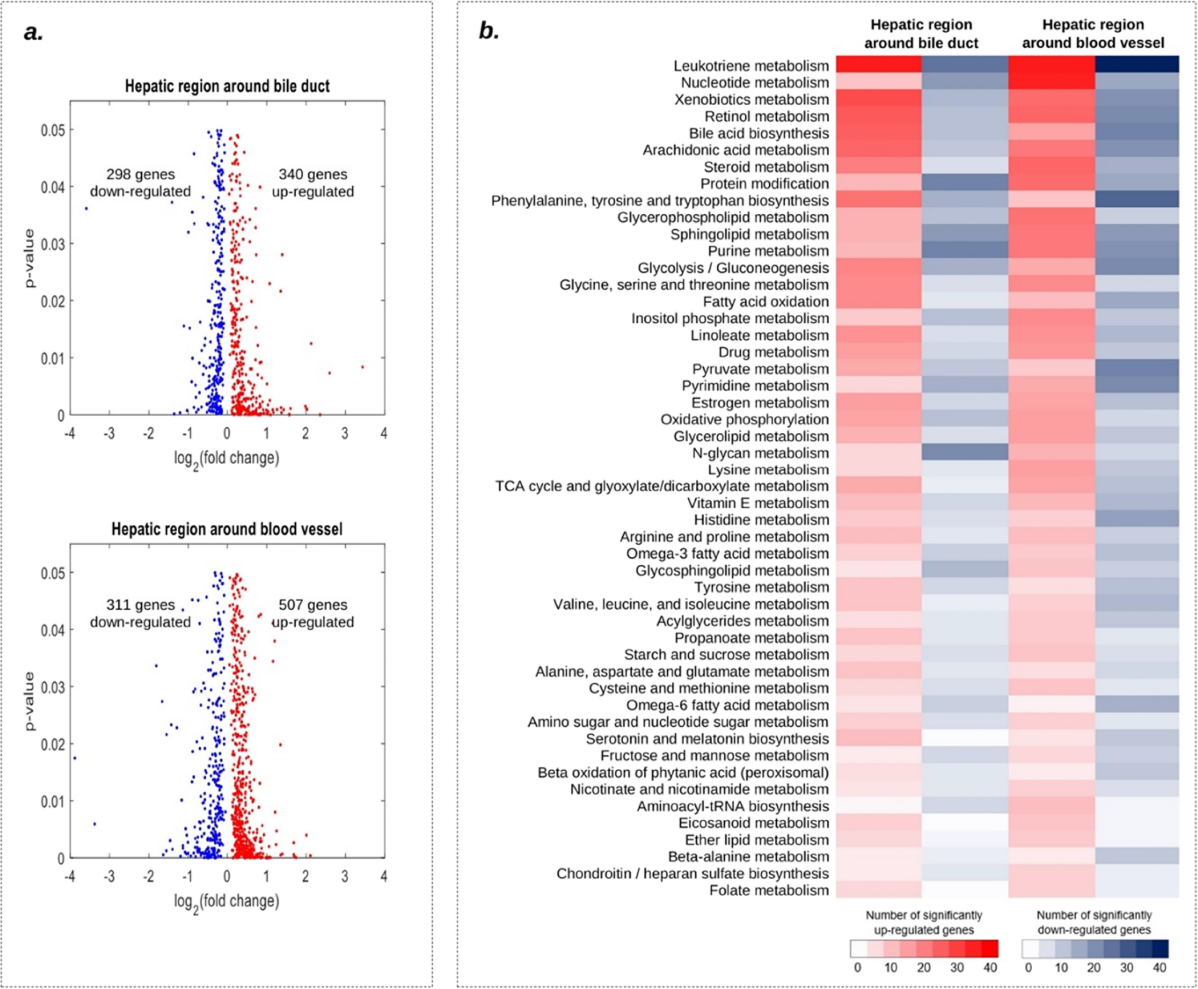
(a) Differential expression analysis for the hepatic region around the bile duct and blood vessel (red, significantly upregulated genes; blue, significantly downregulated genes) and (b) number of significantly up- and downregulated genes in metabolic subsystems.

Fold changes and *p*-values of each gene were calculated by statistically analyzing uninfected and infected gene expressions, and they were mapped to iHepatocytesCovid2406 metabolic network in which 2392 genes were matched with expression data. 340 of them were significantly upregulated (*p*-value < 0.05), and 298 of them were downregulated for the hepatic region around the bile duct (Figure 2a). 507 and 311 genes were significantly up- and downregulated for the hepatic region around blood vessels, respectively. These significantly changed genes were mapped to metabolic pathways in iHepatocytesCovid2406 separately in each group (Figure 2b). Upregulated genes were enriched in leukotriene, nucleotide, xenobiotics, retinol, arachidonic acid, steroid metabolism, and bile acid biosynthesis. Downregulated genes were most significantly enriched in leukotriene, sphingolipid, purine, pyrimidine metabolism, glycolysis/gluconeogenesis, protein modification, and phenylalanine, tyrosine, and tryptophan biosyntheses.

**Figure 3:**
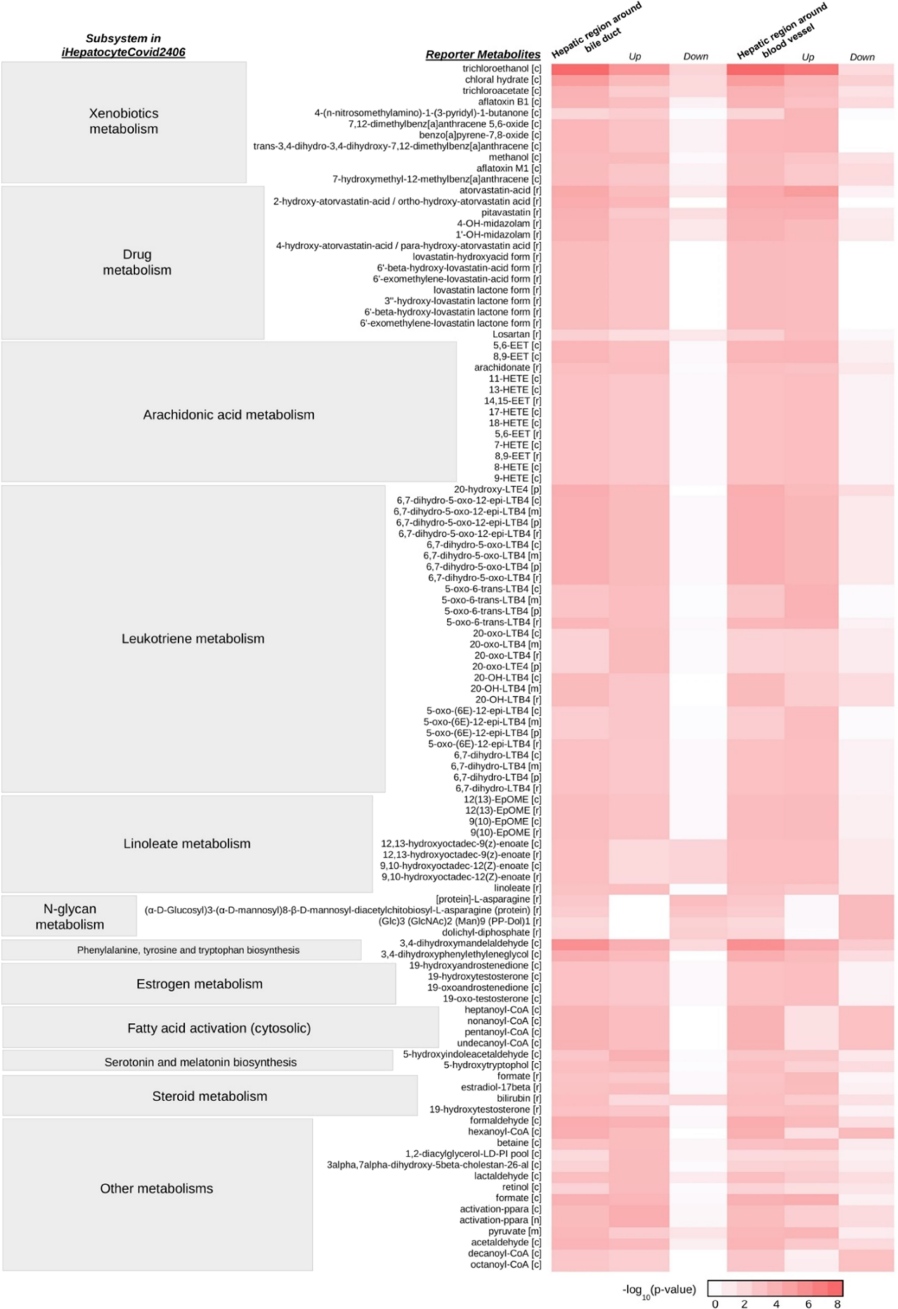
Top reporter metabolites (*p*-value < 0.001) of the hepatic region around the bile duct and blood vessel due to SARS-CoV-2.

Reporter metabolite analysis was performed to elucidate the metabolites around which the most significant transcriptional alterations occurred due to SARS-CoV-2 infection in the hepatic region around the bile duct and blood vessel. The metabolites existing in many reactions were considered currency metabolites (FAD, FADH_2_, H^+^, H_2_O, H_2_O_2_, NADP^+^, NADPH, and O_2_), and they were excluded from reporter metabolites. Trichloroethanol[c], chloral hydrate[c], atorvastatin acid[r], 2-hydroxy-atorvastatin acid/ortho-hydroxy-atorvastatin acid[r], 3,4-dihydroxymandelaldehyde[c], and formaldehyde[c] were predicted as top reporter metabolites (*p*-value < 0.0001) in both hepatic regions (Figure 3). A total of 223 and 242 reporter metabolites (*p*-value < 0.01) were significantly associated with upregulated genes because of SARS-CoV-2 infection in the hepatic region around the bile duct and blood vessel, respectively. The most significant reporter metabolites associated with upregulated genes were involved in xenobiotics, drug, arachidonic acid, leukotriene, and linoleate metabolisms. 62 and 63 reporter metabolites (*p*-value < 0.01), which are related to significantly downregulated genes involved in *N*-glycan, linoleate, steroid, xenobiotics, and drug metabolisms, were observed for bile duct and blood vessel regions, respectively.

A large number of reporter metabolites were involved in arachidonic acid- and leukotriene-related metabolites. Arachidonic acid is an essential fatty acid, a major constituent of cell membrane phospholipids and precursor of bioactive lipids including leukotriene B4 (LTB4) leukotriene C4 (LTC4), leukotriene D4 (LTD4), leukotriene E4 (LTE4), epoxyeicosatrienoic acid, and hydroxyeicosatetraenoic acid.^24^ Since SARS-CoV-2 induces disturbances in leukotriene and arachidonic acid metabolisms, targeting them was offered as novel strategies to control the overall balance of their metabolites in patients with COVID-19.^25,26^ Linoleic acid is the main source of essential *n*-6 polyunsaturated fatty acids, including arachidonic acid. Linoleic acid is also converted to 9(10)-EpOME and 12(13)-EpOME by cytochrome P450.^27^ In addition to leukotriene B4 (LTB4), lipidomic profiling study resulted in increased levels of 9(10)-EpOME and 12(13)-EpOME in COVID-19 patients.^28^ Bilirubin and betaine were determined as reporter metabolites in our analysis. Bilirubin level is a marker of liver damage and is increased in patients with severe COVID-19.^29^ Reduced levels of betaine in individuals with asthma or COVID-19 demonstrated that betaine might serve as a marker for gut health in COVID-19.^30^

### Hepatocellular Rewiring Metabolism in COVID-19 Infection

The hepatocyte metabolic fluxes at an uninfected state were estimated by using the iHepatocytes2434 metabolic network (Supplementary data 2). Maximization of cellular biomass was defined as the objective function in the linear optimization, and subsequently, minimization of metabolic fluxes was applied in quadratic optimization to achieve maximum biomass with minimum flux distribution. Maximum biomass flux resulted in 2.60 mmol/(gDW h) at the uninfected (normal physiological) state. 1854 metabolic reactions have nonzero measurable fluxes [flux value > 10^–6^ mmol/(gDW h)].

**Figure 4:**
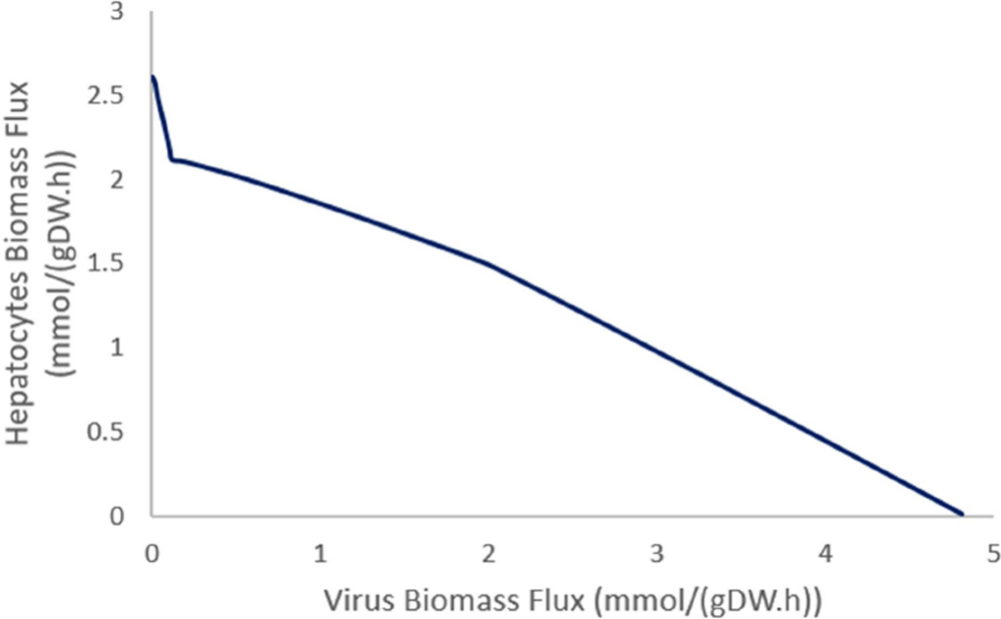
Hepatocytes and virus biomass change during COVID-19 infection.

To elucidate possible hepatocellular rewiring metabolism due to COVID-19 infection, metabolic reactions that exist only in iHepatocytes2434 (56 metabolic reactions) were added to iHepatocytesCovid2406. In the case of infection, the uninfected liver-specific metabolic network should switch to the infected network, and reactions existing only in the uninfected network model must reduce their fluxes to zero. Since there are 10 reactions with nonzero measurable fluxes (flux value > 10^–6^ mmol/(gDW h)) among 56 reactions, their fluxes were gradually reduced to zero by employing MOMA approach to elucidate metabolic alterations due to COVID-19 infection. These gradual reductions (to zero flux) resulted in viral biomass growth by increasing its flux value from 0 to 0.1168 mmol/(gDW h) and a decrease in the hepatocyte biomass flux [2.6080 to 2.1163 mmol/(gDW h)]. These reflect virus growth by hijacking host cell metabolism and achieving the exploitation of host cell metabolites for its replication. Then, the VBOF flux was further increased until zero flux of hepatocyte biomass flux. It approaches zero at 4.85 mmol/(gDW h) of viral biomass flux (Figure 4). This simulation may shed light on how hepatocytes could be exploited by the SARS-CoV-2.

**Figure 5:**
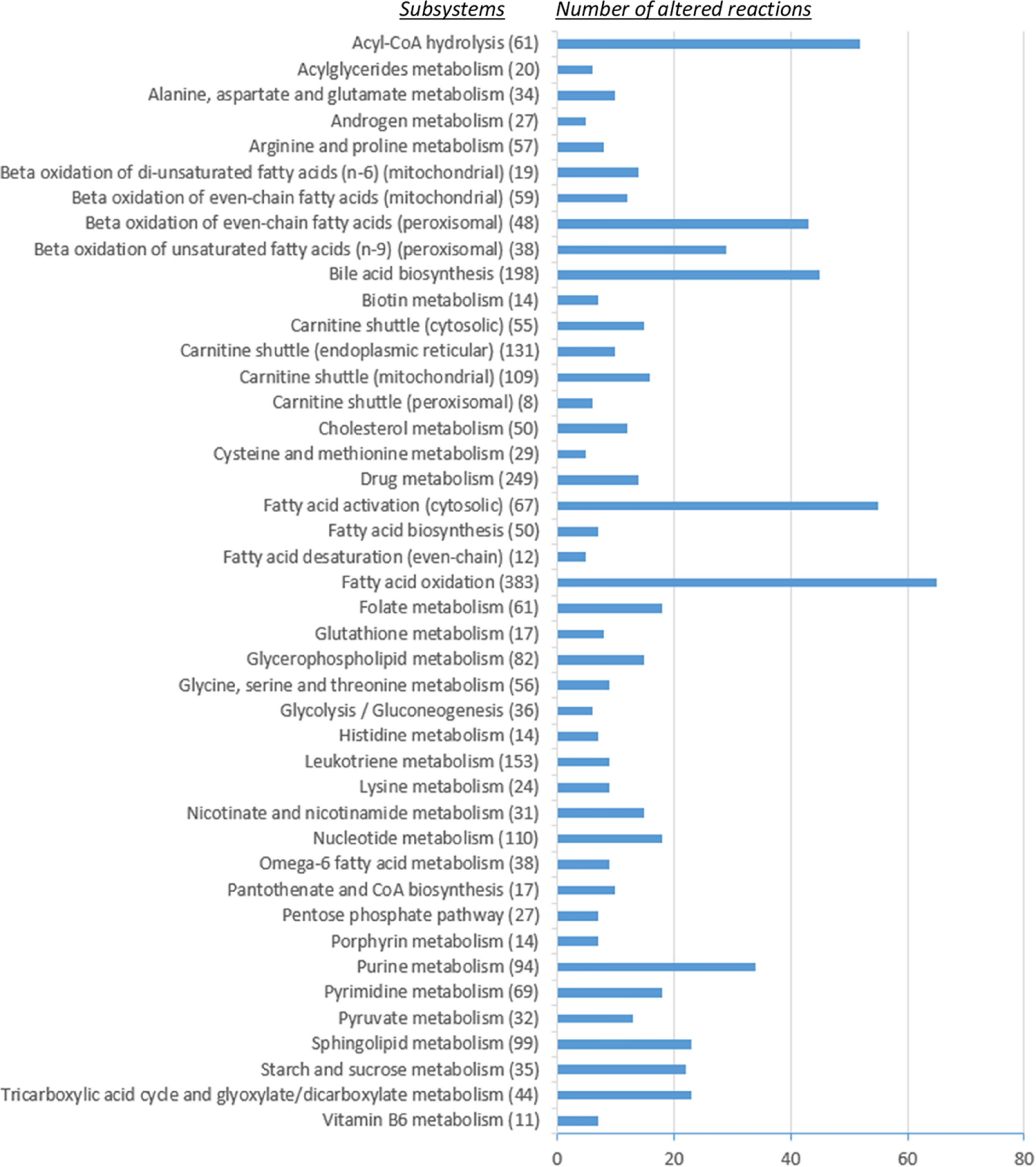
Number of altered reactions in subsystems due to COVID-19 infection (the value in parentheses denotes the total number of reactions in the related subsystem).

Not only the changes in hepatocellular biomass and VBOF but also the flux results can be investigated to understand metabolic shifts due to SARS-CoV-2 infection in the liver. The metabolic fluxes estimated at 2.00 mmol/(gDW h) virus biomass flux were compared with the related uninfected fluxes, and the fold change was computed for reaction fluxes to analyze the number of altered reactions in each metabolic subsystem. In addition to activated and inactivated reactions, altered reactions were assumed to have a fold change value greater than 2 or less than 0.5. The most altered reaction fluxes were accumulated in subsystems of transport and exchange/demand reactions (437 and 204 reactions, respectively). Apart from them, the highest number of altered reaction fluxes were related to fatty acid metabolism (Figure 5). Fatty acids are building blocks of lipids and constitute the major component of the cell membrane. Viruses alter the fatty acid metabolism of the host cell by interacting with them throughout the entrance to the cell and the infection period.^31^ Viral membranes are synthesized by reprogramming of host lipid metabolism for the benefit of the virus. In the plasma of patients with severe pneumonia by SARS-CoV-2, an altered fatty acid profile including a reduction in total fatty acids, phospholipids, and nonesterified fatty acids was observed.^32^ Similar to fatty acids, many reactions were estimated to be altered in glycerophospholipid, sphingolipid, and cholesterol metabolisms which are required for the viral envelope.^33^

Bile acids are synthesized from cholesterol in the liver and play critical roles in lipid metabolism, glucose metabolism, and immune system development.^34^ Together with several reactions in cholesterol metabolism, a significant number of reactions in bile acid biosynthesis were altered due to SARS-CoV-2 (Figure 5). High levels of serum bile acids were monitored in COVID-19 patients.^35^ Carnitine shuttle subsystems have a considerable amount of altered flux due to COVID-19 infection (Figure 5). Carnitine plays an essential role in energy production and fatty acid metabolism. Unlike short- and medium-chain fatty acids, long-chain fatty acids are esterified with carnitine and transported by carnitine shuttle into mitochondria for β-oxidation.^36^ Metabolomics study of COVID-19 patients resulted in altered metabolites involved in carnitine metabolism.^37^ Folate metabolism is critical for nucleotide and glutathione syntheses. They include several altered reaction fluxes estimated in computational analysis (Figure 5). The transcriptional and metabolomic analyses demonstrated that SARS-CoV-2 rewires folate and one-carbon metabolism for its replication.^38^

### Reaction Knockout Analysis

**Figure 6:**
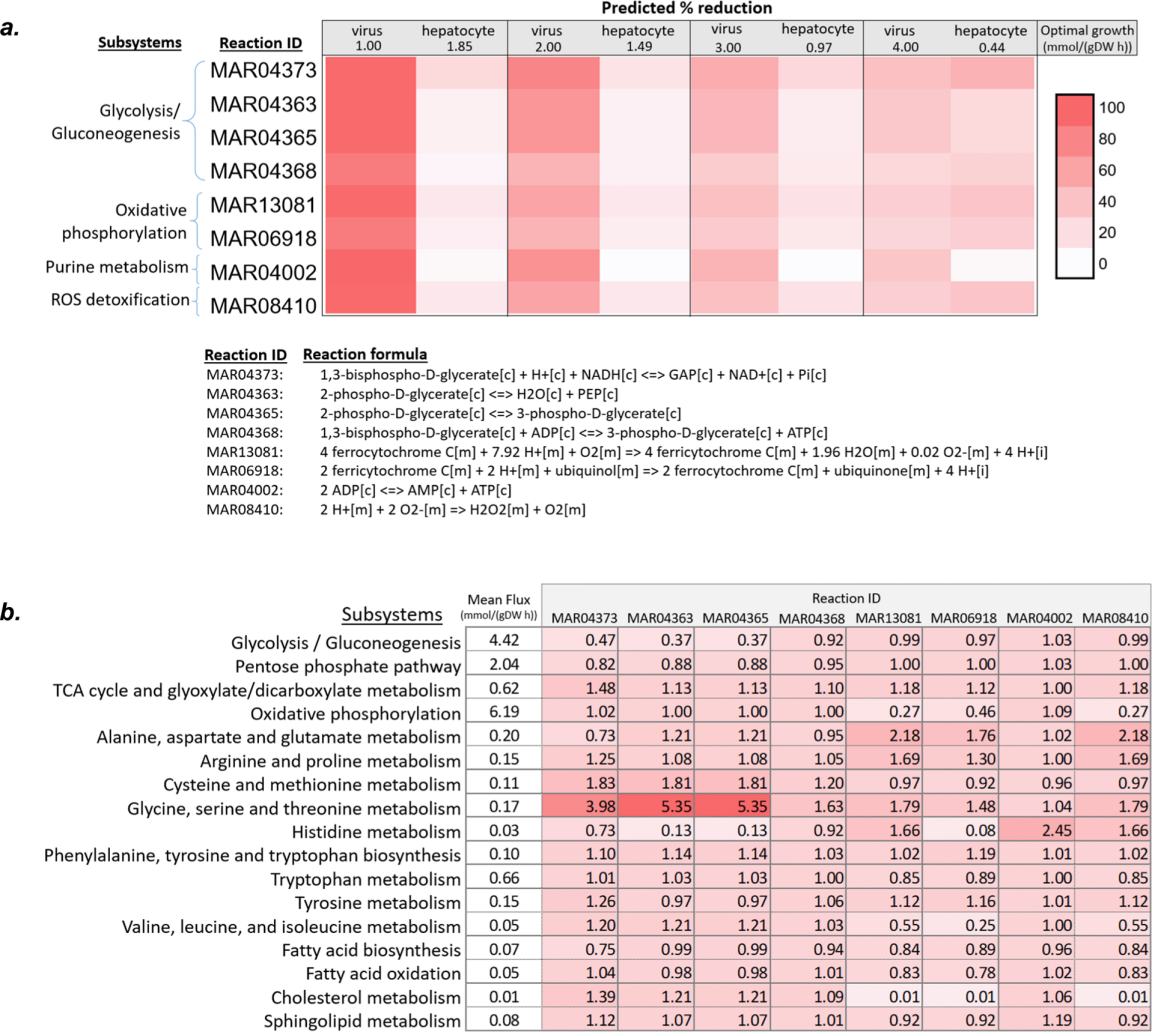
(a) Effect of knockout reactions on virus and hepatocyte growth and (b) fold change in mean of the subsystem fluxes due to knockout of related reaction at 2 mmol/(gDW h) optimal virus growth.

Knockout of a reaction in the host metabolism can limit viral production, and the inhibition of the enzymes catalyzing such reactions gives rise to potential therapeutic strategies against COVID-19.^20,39^ Investigation of such reactions was carried out by reaction knockout analysis at virus growth rate (1, 2, 3, and 4 mmol/(gDW h)) (Figure 6a). The knockout of four metabolic reactions (MAR04020, MAR00592, MAR00610, and MAR00736) from the iHepatocytesCovid2406 metabolic network inhibited both virus and hepatocyte biomass at all stages. Therefore, targeting these reactions is not a good approach to combat the SARS-CoV-2 virus due to inhibition of liver biomass, as well. However, if knockout of a reaction inhibits the viral biomass more than liver biomass, targeting the corresponding reaction provides an alternative strategy.

The knockout of several reactions from the iHepatocytesCovid2406 metabolic network affected the inhibition of host and virus production at different levels at different optimal growth states. The higher percent of reductions was observed in virus growth at 1 mmol/(gDW h), and six of them completely eliminated virus biomass in this optimal state. These computational results show that knockout of the same reaction in an early stage of the infection inhibits viral production higher than that of the late stage. Knockout of the four reactions (MAR04373, MAR04363, MAR04365, and MAR04368), which are involved in glycolysis and the entrance to the tricarboxylic acid (TCA) cycle, increased mean reaction fluxes in amino acids and lipid metabolisms and demonstrated metabolic shift (Figure 6b). The knockout of these reactions remarkably lowered the viral production and were suggested as promising drug targets in the integrated genome-scale analysis of SARS-CoV-2 with human lung cell and bronchial epithelial cells.^39,40^ Glycolysis, which makes crucial substrates and energy contribution for SARS-CoV-2 replication, is dysregulated in COVID-19 patients, and targeting glucose metabolism may help to develop therapeutical strategies.^41,42^ Especially, the regulation of glucose metabolism plays a pivotal role in diabetic patients with COVID-19.^43^

Three mitochondrial reactions in oxidative phosphorylation (MAR13081 and MAR06918) and ROS detoxification subsystems (MAR08410) were estimated to be critical for viral growth. SARS-CoV-2 infection causes development of oxidative stress due to shift in redox homeostasis toward excessive ROS production.^44^ Oxidative stress contributes to immune dysregulation, inflammation, apoptosis, and organ dysfunction. Antioxidant therapies were suggested to attenuate oxidative stress in the treatment of COVID-19.^45^ The knockout of oxidative stress-related reactions resulted in decreased mean reaction fluxes in fatty acid, cholesterol, and sphingolipid metabolisms and enhanced mean fluxes in the TCA cycle and glyoxylate/dicarboxylate metabolism (Figure 6b).

The inhibition of adenylate kinase reaction (MAR04002), which reversibly catalyzes interconversion of ADP to AMP and ATP, was also identified to be critical for viral production via integrated computational analysis of SARS-CoV-2 and liver metabolism (Figure 6a). The guanylate kinase converting ATP and GMP to ADP and GDP was essentially required for viral growth and identified as a potential target for antiviral therapies against SARS-CoV-2 in the human macrophage model,^22,23^ the human lung cell metabolic network,^39,46^ and the human bronchial epithelial cell model.^40^ In the present study, knockout of guanylate kinase reaction (MAR04020) completely inhibited both virus and hepatocyte biomass, and hence the corresponding reaction was not regarded as a potential therapeutical target in SARS-CoV-2-infected liver metabolism. These results show that nucleotide metabolism, regulating pools of purines and pyrimidines, is a key target in viral infections, and organ-specific treatment strategies should be taken into consideration in antiviral research studies.^47^

### Nutrient Supplementation for SARS-CoV-2 Treatment

**Figure 7:**
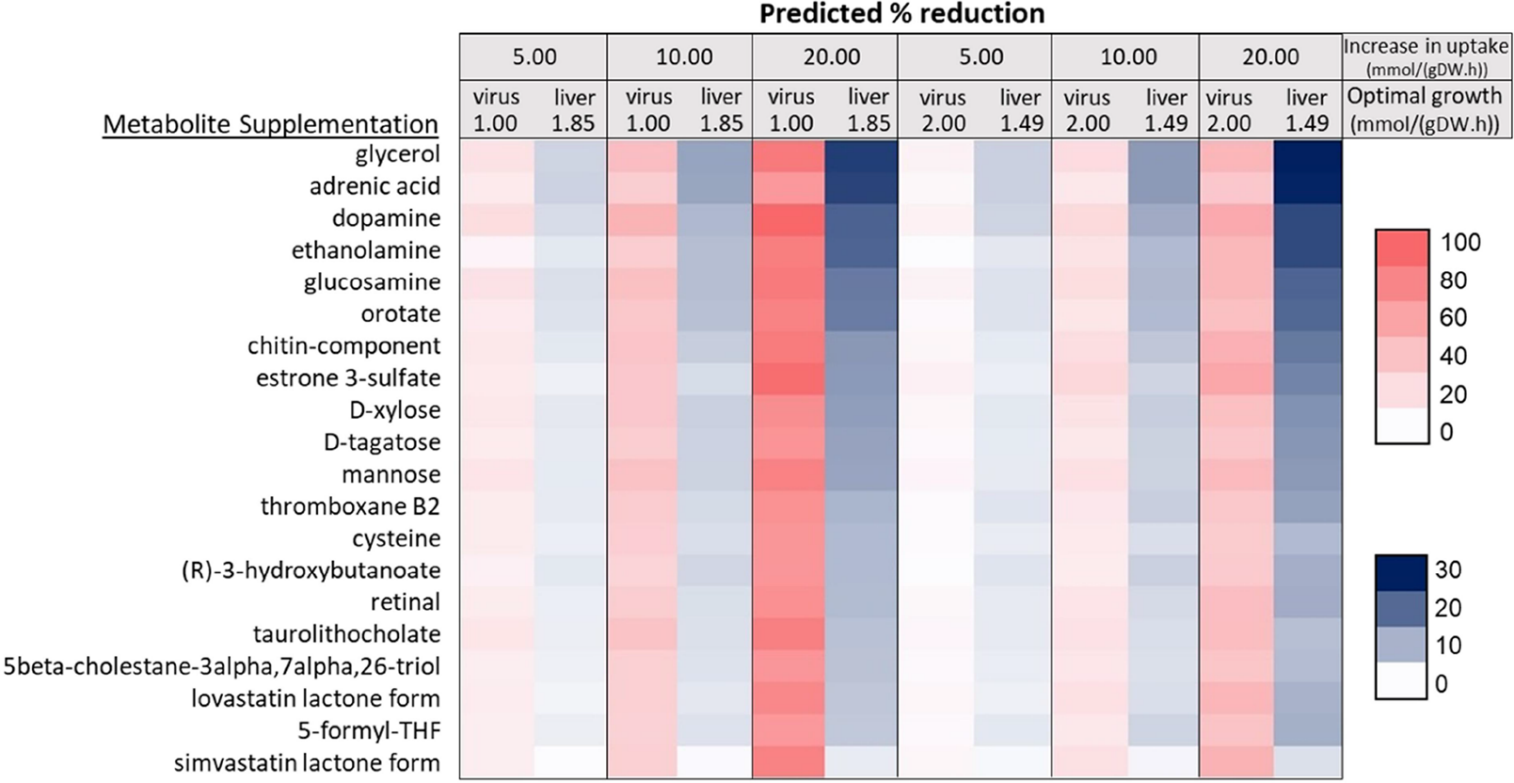
Effect of metabolite supplementation on virus and hepatocyte growth.

Nutrients play an important role in immunomodulation and contribute to mitigation of SARS-CoV-2 infection.^48^ Supplementation of specific nutrients such as vitamins, folate, and *N*-acetylcysteine help to prevent and reduce viral infection. Generated SARS-CoV-2-infected liver-specific genome-scale metabolic network (iHepatocytesCovid2406) was used for the prediction of such nutrients or metabolites. The uptake fluxes of each metabolite available in exchange reactions were increased with the amount of 5, 10, and 20 mmol/(gDW h) at 2 mmol/(gDW h) optimal virus growth, and alterations in virus biomass and hepatocyte biomass were evaluated. If specific metabolite supplementation results in a greater reduction in viral biomass than hepatocyte biomass, then the corresponding metabolite may hold potential for treatment. Several metabolite supplementations were computationally identified to have an inhibitory effect on the viral biomass (Figure 7). Supplements at 1 mmol/(gDW h) optimal virus growth have a higher impact on virus inhibition than those at 2 mmol/(gDW h) optimal virus growth. This result demonstrates that early nutritional interventions are more effective in combating SARS-CoV-2 infection.^49^ Additionally, an enhanced amount of supplements leads to increased reduction in the viral biomass (Figure 7).

Several studies in the literature demonstrated that the supplements used in this study like dopamine, glucosamine, D-xylose, cysteine, (*R*)-3-hydroxybutanoate, lovastatin, and simvastatin have the potential to develop therapeutic strategies to fight SARS-CoV-2.^50−62^ Dopamine is an important neurotransmitter and required for proper functioning of the body including movement, memory, and motivation. Administration of dopamine reduced the viral replication in CaLu-3 human lung epithelial cells and virus-based cytopathic effect in Vero E6 epithelial cells infected with SARS-CoV-2.^50,51^ Glucosamine is a common dietary supplement that promotes joint health. Its administration to infected Calu-3 cells and mice inhibited SARS-CoV-2 replication *in vitro* and *in vivo*, respectively.^52^ In a large population cohort study, the use of habitual glucosamine was connected with decreased hospitalization and mortality due to COVID-19.^53^ The probable therapeutic use of D-xylose was suggested for the treatment of COVID-19 patients.^54^*N*-acetylcysteine is widely used as a health supplement and precursor of cysteine. *N*-acetylcysteine was proposed as both in potential treatment and prevention against SARS-CoV-2 in different studies.^55−57^ (*R*)-3-hydroxybutanoate is a ketone body that provides an alternative carbon source for the cellular metabolism. Ketogenic diet is recommended as nutritional therapy for the treatment of COVID-19 by restoring perturbed energy metabolism and redox state.^60−62^ Lovastatin and simvastatin are lipid-lowering medications and belong to the statin class exhibiting anti-inflammatory, antithrombotic, and immunomodulatory features.^63^ Lovastatin administration in COVID-19 patients led to a significant decrease in the duration of hospitalization in intensive care unit.^58^ Simvastatin treatment lowered viral replication and lung injury in transgenic mice *in vivo* and viral inflammation in different tissues *in vitro.*(59)

## Methods

### Reconstruction of Host–Virus Genome-Scale Metabolic Model

Host–virus genome-scale metabolic model was reconstructed by integrating transcriptome data and Human1 metabolic network.^11^ Transcriptome data (GSE193330) were obtained from Gene Expression Omnibus.^64^ GSE193330 includes transcriptomic analysis from uninfected and SARS-CoV-2 infected hepatocytes collected from liver-on-a-chip (LoC) technology that was designed to represent the hepatic region around the bile duct (ibd-LoC) and blood vessel (bv-LoC).^65^ The integration was performed by tINIT algorithm which generates functional context-specific GEMs by using omics data and predefined metabolic tasks.^15^ The resulting GEMs must perform these tasks, which are essential for all human cells. The algorithm was implemented in the RAVEN Toolbox^66^ in MATLAB (2020a) using the Gurobi and IBM Cplex Optimizer.

Along with gene expression and generic human metabolic model (Human1), 57 essential tasks were used to generate GEMs for uninfected hepatocytes.^11^ When considering the viral infection, an additional virus biomass reaction is required to analyze host–virus interactions. Virus biomass reaction was obtained from Leonidou et al. (2023)^40^ and added to both Human1 and essential tasks for the extraction of virus infected hepatocyte GEMs by using the tINIT algorithm. Gene expression data were given as TPM (transcripts per million reads) normalized with three replicates for each uninfected and infected hepatocyte. Mean values of TPM were calculated, and a cutoff value of 1 was selected in the algorithm. Two GEMs (ibd_uninfectedGEM and bv_uninfectedGEM) were generated for uninfected hepatocytes (around the bile duct and blood vessel), and two GEMs (ibd_infectedGEM and bv_infectedGEM) were generated for SARS-CoV-2 infected hepatocytes. Then, RAVEN-compatible model structures were converted into COBRA-compatible model structures by using “ravenCobraWrapper” function. The uninfected and infected GEMs were merged via “mergeTwoModels” function in the COBRA Toolbox^67^ in MATLAB (2020a) to generate hepatocyte-specific iHepatocytes2434 and iHepatocytesCovid2406 GEMs for uninfected and infected states, respectively. Metabolic task performance of the generated GEMs was evaluated based on 257 metabolic tasks by using the “checkTasks” function in the RAVEN Toolbox.^11^

### Reporter Metabolite Analysis

Reporter metabolites are defined as the metabolites around which the most significant transcriptional alterations exist due to perturbations.^68^ In this study, COVID-19 infection is regarded as the perturbation causing transcriptional changes in the hepatocyte-specific metabolic network. The SARS-CoV-2 infected samples of the hepatic region around the bile duct and blood vessel were statistically compared with related uninfected samples. In addition to fold changes, *p*-values were calculated by using the *t*-test for each gene. Then, they were integrated with iHepatocytesCovid2406 in the prediction of reporter metabolites by the “reporterMetabolites” function in the RAVEN toolbox.

### FBA

FBA was applied to estimate the metabolic reaction fluxes in the uninfected hepatocellular metabolic network (iHepatocytes2434) by using IBM Cplex Optimizer in MATLAB.^7^ FBA assumes the metabolic network at steady state and employs a linear optimization approach with the defined objective function and a set of constraints based on reaction stoichiometry and flux limitations. Uptake and secretion rates for the uninfected condition were obtained from previous hepatocyte GEM study^8^ and defined as constraints by setting upper and lower flux bounds. Maximization of the biomass reaction was selected as an objective function in the linear optimization. Due to multiple optima possibility, minimization of the squared sum of all fluxes was performed as the second objective function in quadratic programming.^69^ The maximum value of biomass reaction flux obtained from the first optimization (linear optimization) was fixed at the second optimization to predict the minimum sum of flux distribution in the uninfected (normal physiological) state.

### MOMA

MOMA was applied for the analysis of host and SARS-CoV-2 interaction.^70^ MOMA predicts an optimal solution by using quadratic programming and performing distance minimization between uninfected and infected flux spaces. Therefore, it hypothesizes metabolic networks’ response to perturbation with minimum flux change. Here, SARS-CoV-2 infection is regarded as a perturbation. Uninfected reaction fluxes estimated from iHepatocytes2434 were mapped to related reaction fluxes in iHepatocytesCovid2406 to represent initial values. The reactions that are available in iHepatocytes2434 but not in iHepatocytesCovid2406 were merged with the iHepatocytesCovid2406 metabolic network. The fluxes of these reactions were gradually decreased to zero by arranging upper and lower bounds to elucidate possible hepatocellular rewiring metabolism due to the COVID-19 infection. Then, the virus biomass reaction flux was gradually increased by changing its upper and lower bounds by applying the MOMA approach.

### Reaction Knockout Analysis

The lower and upper bounds of each reaction (excluding exchange and transport reactions) were systematically set to zero so as to point out the effect of the related reaction knockout on the viral biomass and hepatocellular biomass. MOMA approach was applied to simulate the minimum flux response to individual reaction knockout. If the deletion of a specific reaction inhibits virus biomass growth, this reaction is regarded as critical for SARS-CoV-2. Antiviral strategies were suggested to combat SARS-CoV-2 infection by comparing the changes of virus and hepatocellular biomass.

### Nutrient Supplementation Analysis

Supplementation of specific metabolites or nutrients may result in the inhibition of virus biomass. To investigate such metabolites, the uptake flux of each metabolite was increased through exchange reactions in the iHepatocytesCovid2406 metabolic network. MOMA approach was used in the computation. The changes in virus and hepatocyte biomass were examined due to specific metabolite supplementation.

## Conclusions

Integrated host–virus GEMs provide an estimation of promising therapeutic targets by taking a comprehensive analysis of the metabolic interactions between viruses and their host cells into account. The comparison of GEMs, generated from uninfected and infected samples, gives clues about virus-induced metabolic alterations, and treatment strategies are suggested to inhibit or attenuate viral infection. Here, context-specific GEMs were reconstructed for uninfected and SARS-CoV2 infected hepatocytes based on transcriptome data. In line with experimental studies in the literature, reporter metabolites and altered reactions revealed metabolic reprogramming in a variety of subsystems including arachidonic acid, leukotriene, fatty acid, glycerophospholipid, sphingolipid, cholesterol, and folate metabolisms, bile acid biosynthesis, and carnitine shuttle among others. Subsequently, reaction knockout analysis predicted critical reactions for viral replication. An increase in the uptake of several metabolites such as dopamine, glucosamine, D-xylose, and (*R*)-3-hydroxybutanoate simulated a decline in the virus production. Consequently, integrated host–virus GEMs can help the development of novel therapeutic strategies to combat viruses. Additional computational and experimental research is still needed to fully understand SARS-CoV-2 interactions with various organs in the human body and comorbidities including neurodegenerative diseases, hypertension, cancer, diabetes, and obesity. Clinical trials are also required for computational strategies, and taking full advantage of them can accelerate experimental drug target studies, especially in the sudden emergence of viral diseases.

## Supporting Information Available

iHepatocytes2434 and iHepatocytesCovid2406 metabolic models (ZIP)
Uninfected hepatocytes metabolic flux distribution (XLSX)

## Abbreviations

9,10-EpOME: 9,10-epoxyoctadecenoic acid
12,13-EpOME: 12,13-epoxyoctadecenoic acid
EET: epoxyeicosatrienoic acid
FBA: flux balance analysis
GEM: genome-scale metabolic model
HETE: hydroxyeicosatetraenoic acid
LTB4: leukotriene B4
LTC4: leukotriene C4
LTD4: leukotriene D4
LTE4: leukotriene E4
MOMA: minimization of metabolic adjustment
ROS: reactive oxygen species
SARS-CoV-2: severe acute respiratory syndrome coronavirus 2
TCA: tricarboxylic acid
tINIT: task-driven integrative network inference for tissues
VBOF: virus biomass objective function
